# High-Intensity Ultrasound-Assisted Extraction of Pectin from Mango Wastes at Different Maturity

**DOI:** 10.1155/2022/4606024

**Published:** 2022-07-15

**Authors:** Ramiro Torres-Gallo, Sulibeth Bayuelo-Bonilla, Luz Carpio-Ortiz, Genisberto Barreto-Rodríguez, Diego F. Tirado

**Affiliations:** ^1^Departamento de Ingeniería Agroindustrial, Facultad de Ingeniería, Universidad del Atlántico, Km 07, Vía a Puerto Colombia, 232527 Barranquilla, Colombia; ^2^Dirección Académica, Universidad Nacional de Colombia, Sede de La Paz, La Paz 202017, Colombia

## Abstract

Valorisation of food processing by-products is a welcome and developing area. The mango processing industry produces 40% to 60% of the fruit as solid waste, in which components of industrial interest, such as pectin, are lost. This study reports on energy-efficient high-intensity ultrasound-assisted extraction (HIUAE) to extract pectin from mango peels. The analysis considered the ripening stage of the fruit (0, 2, and 4), HIUAE frequency (37 kHz and 80 kHz), and extraction time (20 min, 25 min, and 30 min). Extractions of pectin from mango peels with HIUAE have been fairly studied. However, this work differs from those studies in including mango maturity grade as a factor. Pectin extraction yields ranged from 13% to 30%, with no influence (*p* > 0.05) of time, and the highest yields were obtained at the lowest maturity stage (0) and lowest frequency (37 kHz). This latest condition (37 kHz) also yielded pectin with the highest gel strength, purity, and quality. This work demonstrated that the mango maturity stage influenced pectin extraction yield. Ultrasound-assisted extraction of pectin from mango peels could be an efficient approach toward waste valorisation and extraction of pectin with high yield and good quality attributes for the food industry.

## 1. Introduction

The circular economy focuses on resource efficiency and maximum utilization of waste. In recent years, this concept has become a promising alternative for the food industry, as it converts industrial waste into products with high biological value and, at the same time, reduces the impact on the environment [1]. Bioeconomy is a rapidly growing industry, highly liked to the circular economy [2], which is consolidated as an essential driver of sustainable development for Colombia, with a territorial approach, based on the use of biomass.

Mango (*Mangifera indica L.*) is the most consumed fresh fruit in the world due to its high nutritional and bioactive properties, which is why it is known as the “king of fruits” [3]. The mango processing industry produces a large amount of solid waste such as peels, pomace, and seeds, representing 40% to 60% of the fresh fruit. Peels alone account for half of this waste [4]. These industrial by-products are usually burned or left in the open air for decomposition [5]; none are currently used for commercial purposes, even though they could contain compounds of industrial interest such as pectin [6].

Pectin is the most widely used polysaccharide in the food industry [7]. Pectin is a plant cell wall polysaccharide, consisting mainly of D-galacturonic acid, followed by D-galactose and L-arabinose [8]. Raw materials such as citrus peels and apple pulp have been used for pectin production [9], although mango also contains pectin in its peel [10]. Traditionally, pectin extraction uses an acid solution and heat [8]; however, emerging technologies such as high-intensity ultrasound-assisted extraction (HIUAE) can improve the efficiency of the process [11].

The conventional extraction method requires long processing times, high energy requirements, and high solvent consumption with a high amount of wastewater generation [12]. Due to these drawbacks related to conventional extraction, emerging technologies such as HIUAE arise, which combines an assisted extraction in which ultrasound provides external energy to improve mass transfer [13].

HIUAE is more beneficial due to low energy consumption, shortened treatment time, less solvent usage, increased safety of the operators, and increased yield [7]. Thus, in this research, pectin was extracted from mango peels as a function of the maturity of the fruit by using HIUAE to assess its potential utilization as an alternative source of commercial pectin production.

## 2. Materials and Methods

### 2.1. Materials

Mangoes used in this study were of the hilaza variety collected at a local market in Barranquilla (Atlántico, Colombia). Three maturity stages (0, 2, and 4) were categorized by colour according to the Colombian Technical Standard 5139 [14]. Mangoes were disinfected with sanitizer and washed with water. The mango pulp was first analysed. For this, the concentration of soluble solids was determined using a refractometer (model HI96801, Hanna Instruments, United States), and the titratable acidity was obtained by the volumetric method. The peel was separated from the fruit using a stainless-steel knife, blanched at 80°C ± 2°C for 10 min, and then cut into 1 cm × 1 cm squares to increase the surface area. It is well known that blanching helps to peel fruits; also, it inactivates enzymes, inhibits microbial growth, and removes pesticide residues.

### 2.2. Pectin Extraction

The process of pectin extraction was based on the method carried out by Esparza-Merino et al. [15]. For this, samples from each maturity stage were added to a hydrochloric acid solution at pH 1.5 and a sample/medium ratio of 1 : 10. They were then treated in a high-intensity ultrasound bath (Elmasonic®, P30H 3L, Germany) at different frequencies (37 kHz and 80 kHz) and extraction times (20 min, 25 min, and 30 min) using distilled water at 30°C ± 1°C as a transmission medium. Acid hydrolysis was carried out with HCl at 80°C for 1 h under constant agitation, using 96% ethanol/water at a 1 : 1 volumetric ratio. The obtained gel was then separated with a filter cloth and purified with 50% ethanol. After that, the gel was dried using a tray dehydrator (WESTON®, United States) at 40°C until a constant weight was achieved. Finally, the resulting material was ground in an electric mill (CGoldenwall, United States). The yield was determined as the ratio of the weight of dry pectin to the weight of mango peels on a dry basis, considering Equation 1.

(1) $$\begin{array}{c} \text{Yield }\left(\%\right)=\frac{\text{Pectin dry weight }\left(\text{g}\right)}{\text{ Sample dry weight }\left(\text{g}\right)}\times 100. \end{array}$$

### 2.3. Physicochemical Characteristics of Pectin

The equivalent weight of the extracted pectin was determined by titration as described by Siddiqui et al. [7]; ash content was determined by the muffle burn method AOAC 942.05 [16]; and free acidity was measured as titratable acidity, and the results were expressed as per cent of citric acid.

To determine the degree of esterification, methoxy groups, and galacturonic acid, 5 g pectin was mixed with a solution containing 100 mL of 60% ethanol and 5 mL of HCl (37% *w*/*w*). Subsequently, the mixture obtained was homogenized at room temperature for 10 min. The residues were filtered and washed six times with 15 mL of the ethanol/HCl solution (1 : 1) and then with 20 mL of ethanol. Finally, the residues were dried at 40°C for 1 h. One-tenth of the dried sample was mixed with 2 mL of ethanol and 100 mL of distilled water. The mixture was stirred until the pectin was completely dissolved. The volume was titrated with 0.5 N NaOH (*V*_1_) and phenolphthalein as an indicator. Then, 20 mL of 0.5 N NaOH was added and allowed to stand for 15 min. Afterwards, 20 mL of 0.5 N HCl was added, and the mixture was stirred until the pink colour disappeared. The final volume was titrated with 0.5 N NaOH (*V*_2_) and phenolphthalein as an indicator. The degree of esterification was determined as follows by Equation 2

(2) $$\begin{array}{c} \text{Degree of esterification }\left(\%\right)=\left[\frac{V_{2}}{V_{1}+V_{2}}\right]\times 100\%. \end{array}$$

The percentages of methoxy groups and galacturonic acid were determined as follows by Equation 3 and Equation 4, respectively.

(3) $$\begin{array}{c} \text{Methoxy groups }\left(\%\right)=\frac{V_{1}\times N\times m_{\text{eq}}}{w}\times 100\%, \end{array}$$

(4) $$\begin{array}{c} \text{Galacturonic acid }\left(\%\right)=\frac{V_{2}\times N\times m_{\text{eq}}}{w}\times 100\%, \end{array}$$

where *N* is the normality of NaOH, *m*_eq_ is the milliequivalents of methoxy (0.031 in Equation (3)) and galacturonic acid (0.097 in Equation (4)), and *w* is the weight of the sample (g).

### 2.4. Analysis of Infrared Spectroscopy

The analyses were performed on a Fourier transform infrared spectrometer model IRAFFINITY-1 (SHIMADZU®, Japan). For this, a 1.0 mg sample was prepared with potassium bromide (KBr). Transmittance measurements were obtained in a spectral scanning range from 4000 cm^−1^ to 400 cm^−1^ and a resolution of 4 cm^−1^.

### 2.5. Determination of the Degree of Gelation in Mango Jam

To evaluate the degree of pectin gelation, a jam was made with mango pulp and mango peel pectin considering the three maturity stages (0, 2, and 4) and HIUAE frequencies (37 kHz and 80 kHz). Sugar and potassium sorbate were added to the formulation. Jam viscosities were determined in a digital viscometer (NDJ-8S®, China) using No. 4 needles, 1.5 rpm, and 30 s of agitation.

### 2.6. Experimental Design and Statistical Analysis

The experiments were performed under a randomized three-factor design, which included the maturation stage (0, 2, and 4), ultrasound exposure time (20 min, 25 min, and 30 min), and ultrasound frequency (37 kHz and 80 kHz). Analysis of variance (ANOVA) and Tukey's test (5% significance) were performed for the response variables (pectin extraction yield, physicochemical characteristics of the extracted pectin, and degree of gelation in mango jam) using GraphPad 5 software. The experiments were repeated three times. Analyses were performed in duplicate for each replicate (*n* = 3 × 2). Means and standard deviations were calculated and reported for all data.

## 3. Results and Discussions

### 3.1. Mango Maturity Index

Table 1 shows total soluble solids and acidity of mango pulp used in this study at the selected maturity stages. As can be seen in Table 1, there was a proportional relationship between total soluble solids and maturity stage. Conversely, there was a decrease in acidity with increasing mango maturity stage. Both results were normal. The first was associated with the hydrolysis of starch to simple sugars during ripening, while the reduction in acidity was related to the degradation of pectin, cellulose and hemicellulose, and characteristic of climacteric fruits such as mango [17].

### 3.2. Pectin Extraction Yield from Mango Peels

**Figure 1:**
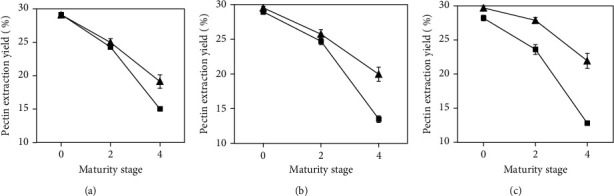
Pectin extraction yield from mango peel as function of maturity stage (0, 2, and 4) at 37 kHz (▲) and 80 kHz (■): (a) 20 min, (b) 25 min, and (c) 30 min.

Figure 1 shows the extraction yield of pectin from mango peels as a function of the maturity stage, extraction time, and HIUAE frequencies. Pectin extraction yields ranged from roughly 13% (maturity stage 4, 80 kHz, and 30 min) to 30% (maturity stage 0, 37 kHz, and 30 min). The latter could be considered as a high yield of pectin extraction, in comparison with data obtained in the literature [18]. Therefore, it was observed that the yields obtained were acceptable and that mango peels could represent an alternative source with great potential for pectin extraction. Additionally, extraction yield results (see Figure 1) showed that time had no statistical influence (*p* > 0.05) on the degree of pectin extraction; therefore, 20 min extraction was chosen for the following experiments. In addition, this was the condition that represented the lowest energy consumption of the process, thus making it much more profitable on a commercial scale [19].

In addition, Figure 1 shows that there was a clear inverse relationship between maturity stage and pectin extraction yield, with the highest yields obtained at the lowest maturity stage. This was due to the lower presence of pectin in the fruit at higher maturity stages. The higher extraction yield was found in maturity stage 0, in which there was less degradation of pectin, cellulose, and hemicellulose [17]. In stages 2 and 4, the mango already had a higher content of the enzymes pectin methylesterase and polygalacturonase, which reduced the pectin content and consequently the firmness of the fruit [20].

Apart from the aforementioned, regardless of the maturity stage, the highest pectin extraction yields were always obtained at the lowest frequency (37 kHz), being much more pronounced at 30 min, conditions (37 kHz and 30 min) at which the highest pectin extraction yields were observed. At 37 kHz, there were no statistically significant differences (*p* > 0.05) between the extraction yields at 20 min and 25 min. An opposite behaviour occurred at a higher frequency, as the lowest yields were obtained at 80 kHz, and these decreased with increasing time, with the lowest extraction yield being found at 80 kHz and 30 min.

The higher pectin extraction yield at a lower frequency could be due to the fact that the lower the frequency the more the interactions between the solvent and pectin as a consequence of the closeness of this frequency to the resonance frequency of the bubbles, so that the bubbles collapsed violently, which was favourable for improving the extraction yields of pectin [21]. On the contrary, at a higher frequency, there was probably a reduction of the cavitation effect caused by the rarefaction cycle of the sound wave, which created a negative pressure that was not sufficient in its intensity to start cavitation; thus, the compression cycle was faster than the microbubble formation time to collapse [22].

The higher yield at the longest exposure time and lowest frequency (30 min and 37 kHz) may be explained by the higher destruction of glycosidic and ester bonds at longer exposure times [23]. This was in agreement with Grassino et al. [24], who obtained a similar behaviour when extracting pectin from tomato residues.

The selection of the extraction method is a key stage in obtaining bioactive compounds. A wide spectrum of techniques is currently available for this purpose, such as maceration, Soxhlet extraction, supercritical extraction, microwave-assisted extraction, pulsed electric field-assisted extraction, high pressure solvent extraction, and HIUAE, of course [12, 25]. HIUAE belongs to the so-called green technologies along with supercritical fluid, microwave, and pulsed electric field extraction. These technologies have a lower impact on the environment in terms of use of organic solvents, time, and energy. HIUA has many advantages, such as relatively short process time, less solvent consumption, and low equipment cost. The disadvantage of using this method is the need to separate the extract from the residue after extraction [12].

### 3.3. Equivalent Weight of Mango Peel Pectin

**Figure 2:**
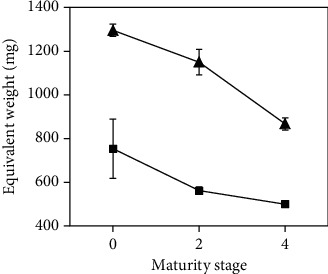
Equivalent weight of pectin extracted from mango peel as function of maturity stage (0, 2, and 4) at 37 kHz (▲) and 80 kHz (■) after 20 min.

The equivalent weight of pectin is an indicator of gel-forming ability. The higher the equivalent weight, the higher the gel strength, provided by the number of galacturonic acid residues in the molecule, which generates higher quality pectin. The equivalent weight of pectin obtained from mango peels in this work was higher at 37 kHz than 80 kHz, decreasing significantly (*p* ≤ 0.05) with the ripening stage, as can be seen in Figure 2. This decrease occurred as a result of the breakage of the galacturonic acid chains during the fruit ripening process, because the number of esterified carboxyl was reduced and converted into free carboxyl, causing a decrease in the equivalent weight of pectin [26]. The equivalent weight of pectin obtained from mango peels in this work at 80 kHz ranged between 500 mg and 754 mg for maturity stages 4 and 0, respectively. On the other hand, at 37 kHz, the equivalent weight was between 867 mg and 1295 mg, for maturity stages 4 and 0, respectively. Therefore, extractions at the lowest frequency resulted in pectin with higher gel strength and consequently higher quality. These values agreed with data reported elsewhere [7, 27].

### 3.4. Chemical Composition of Extracted Pectin

The ash content indicates the purity of the pectin. The lower the ash content, the higher the purity level of the pectin. As shown in Table 2, the ash content in pectin from mango peels after 20 min extraction ranged from 0.4% to 0.8%, being much lower at the lowest frequency. These values were below those reported by Grassino et al. [24], who obtained 1.2%-2.6% ash content in pectin extracted from banana peels under similar extraction conditions to this work. Therefore, our data proved that the purity of pectin extracted from mango peel was higher at a lower frequency (37 kHz).

As can be seen in Table 2, the free acidity of pectin from mango peels increased with the maturity stage at both frequencies, reaching higher values at 80 kHz. The inverse ratio between equivalent weight and free acidity could explain this behaviour [7, 27]. Finally, the results obtained for anhydrous galacturonic acid content ranged from 55% (maturity stage 0 and 37 kHz) to 100% (maturity stage 4 and 80 kHz). These data were similar to those reported by Wang et al. [18] for mango peel pectin obtained by HIUAE.

### 3.5. Degree of Esterification of Mango Peel Pectin

**Figure 3:**
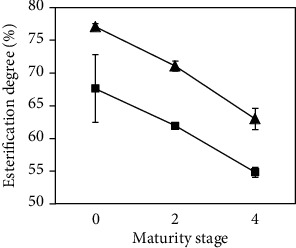
Degree of esterification of extracted pectin after 20 min in relation to maturity stage (0, 2, and 4) at 37 kHz (▲) and 80 kHz (■).

As can be seen in Figure 3, the degree of esterification decreased with the maturity stage at both frequencies, with higher values at 37 kHz. The values obtained for the degree of esterification in pectin extracted at different maturity stages ranged from 55% (maturity stage 4 and 80 kHz) to 77% (maturity stage 0 and 37 kHz). The latter was similar to the results reported by Freitas de Oliveira et al. [28], who obtained values between 77% and 80% when using ultrasound, and was also similar to the results reported by Guandalini et al. [11], with 58%-67% in pectin obtained from mango peels. The lower degree of esterification at higher frequencies was caused by the deesterification of the polygalacturonan chains due to strong acids [29].

### 3.6. Infrared Spectral Analysis of Mango Peel Pectin

**Figure 4:**
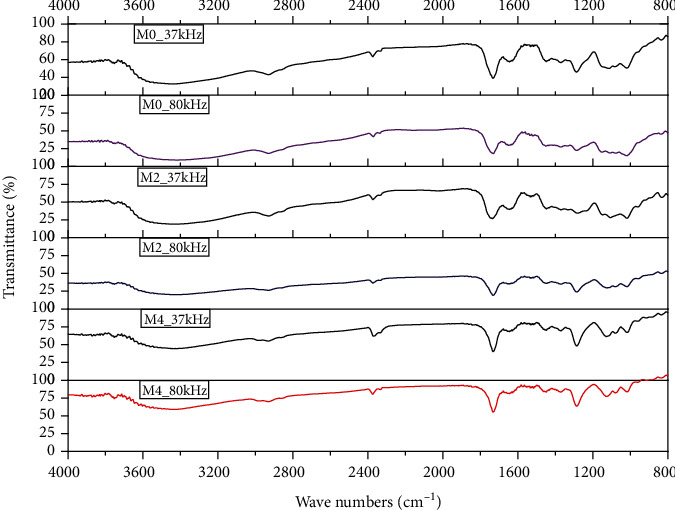
FTIR spectrum of pectin extracted from mango peel after 20 min, different maturity stages (M0, M2, and M4), and ultrasonic pretreatment frequency (37 kHz and 80 kHz).

The remarkable peaks in the FITR spectra of the extracted pectin at different maturity stages and frequencies were symmetrical, as can be seen in Figure 4. As can be seen in Figure 4, the broad and strong absorption band appearing around 3421 cm^−1^ corresponded to the O-H vibration due to inter- and intramolecular hydrogen bonds located in the main chain of galacturonic acid [30]. As Figure 4 shows, this band was broader and less intense in pectin extracted at 80 kHz, due to the reduction in the number of O-H bonds and the appearance of the O-CH_3_ bond vibration band, which was also evident as the maturity stage increased at the same treatment frequency [31].

### 3.7. Viscosity Analysis

**Figure 5:**
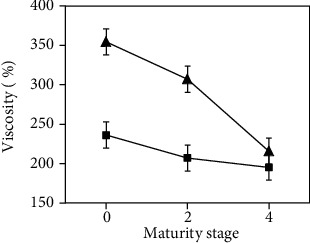
Viscosity of jams made from mango peel pectin extracted after 20 min at different maturity stages (0, 2, and 4) at 37 kHz (▲) and 80 kHz (■).

Figure 5 shows the viscosity analysis of jams made from mango peel pectin. The results show that viscosity decreased with frequency and the ripening stage. These results were similar compared to the equivalent weight and degree of esterification, since these properties influenced the viscosity and the gelling power of pectin [7, 27].

## 4. Conclusions

Colombia's mango production surpassed 300,000 metric tons in 2021, indicating that the country's mango solid waste capacity exceeded roughly 156,000 metric tons last year. As much as 78,000 tons of this residue were peels, none of which are currently used commercially in Colombia [32].

High-intensity ultrasound-assisted extraction (HIUAE) is extensively used in the food industry due to its capability to process with low energy consumption, short times, low solvent usage, high safety of the operators, and high extraction yields. Therefore, HIUAE of pectin from mango peel was investigated in this work. As a result, pectin obtained at the shortest time, lowest maturity stage, and lowest frequency yielded the product with the highest gel strength, purity, and quality. These conditions represent the most viable conditions on a commercial scale.

Ultrasound-assisted extractions of pectin from mango peels with ultrasonication have been fairly studied, as referenced in this work [11, 18, 27]. This study, however, differs from those past works in including mango maturity grade as a factor; and this is a strength, as mangoes are ripened for mango juice, while mango chips need firmer fruits. The maturity stage of the mangoes positively influenced the pectin extraction yield, giving the possibility of using mangoes that do not reach commercial maturity, thus avoiding waste of this portion of the production.

The major advantages of the HIUAE are the significant shortening of the extraction process and its environmentally friendly nature. Therefore, this technique could be used as an efficient procedure for extracting pectin from mango peels or other by-products from the agroindustrial business. Likewise, these techniques help revalue industrial by-products that contribute to environmental damage.

## Figures and Tables

**Table 1:** Soluble solids and acidity of mango according to maturity index.

Physicochemical properties	Maturity stage		
	0	2	4
Total soluble solids (°Brix)	7.5 ± 0.1^a^	12.0 ± 0.1^b^	15.5 ± 0.1^c^
Titratable acidity (% citric acid)	2.0 ± 0.0^a^	1.0 ± 0.0^b^	0.3 ± 0.0^c^

Values are presented as mean ± standard deviation. Different lowercase letters within the same row represent statistically significant differences at a 5% significance (*p* ≤ 0.05).

**Table 2:** Analysis of chemical components of pectin extracted from mango peel after 20 min.

Ultrasound frequency	37 kHz			80 kHz		
Maturity stage	0	2	4	0	2	4
Ash (%)	0.4 ± 0.0	0.4 ± 0.0	0.5 ± 0.0	0.8 ± 0.0	0.8 ± 0.0	0.4 ± 0.0
Free acidity (meq/g)	0.8 ± 0.0	0.9 ± 0.0	1.6 ± 0.0	1.4 ± 0.2	1.8 ± 0.4	2.0 ± 0.0
Methoxy (%)	8.1 ± 0.4	6.6 ± 0.1	6.1 ± 0.6	8.7 ± 0.5	9.0 ± 0.2	7.5 ± 0.0
Anhydrous galacturonic acid (%)	55 ± 2	65 ± 1	74 ± 0	75 ± 1	80 ± 0	100 ± 3

## Data Availability

The data used to support the findings of this study are available from the corresponding author upon request.

## References

[1] Stahel W. R. (2016). The circular economy. *Nature*.

[2] Gomes-Araújo R., Martínez-Vázquez D. G., Charles-Rodríguez A. V., Rangel-Ortega S., Robledo-Olivo A. (2021). Bioactive compounds from agricultural residues, their obtaining techniques, and the antimicrobial effect as postharvest additives. *International Journal of Food Science*.

[3] Kiloes A. M., Azizan F. A., Checco J., Joyce D., Abdul Aziz A. (2022). What do consumers want in fresh mangoes? A systematic literature review. *International Journal of Food Science & Technology*.

[4] Abbasi A. M., Liu F., Guo X., Fu X., Li T., Liu R. H. (2017). Phytochemical composition, cellular antioxidant capacity and antiproliferative activity in mango (Mangifera indica L.) pulp and peel. *International Journal of Food Science & Technology*.

[5] Manhongo T. T., Chimphango A., Thornley P., Röder M. (2021). Techno-economic and environmental evaluation of integrated mango waste biorefineries. *Journal of Cleaner Production*.

[6] Kumoro A. C., Alhanif M., Wardhani D. H. (2020). A critical review on tropical fruits seeds as prospective sources of nutritional and bioactive compounds for functional foods development: a case of Indonesian exotic fruits. *International Journal of Food Science*.

[7] Siddiqui A., Chand K., Shahi N. C. (2021). Effect of process parameters on extraction of pectin from sweet lime peels. *Journal of The Institution of Engineers (India): Series A*.

[8] Pérez J., Gómez K., Vega L. (2022). Optimization and preliminary physicochemical characterization of pectin extraction from watermelon rind (Citrullus lanatus) with citric acid. *International Journal of Food Science*.

[9] Naqash F., Masoodi F. A., Gani A., Nazir S., Jhan F. (2021). Pectin recovery from apple pomace: physico-chemical and functional variation based on methyl-esterification. *International Journal of Food Science & Technology*.

[10] Rojas M. L., Kubo M. T. K., Caetano-Silva M. E., Augusto P. E. D. (2021). Ultrasound processing of fruits and vegetables, structural modification and impact on nutrient and bioactive compounds: a review. *International Journal of Food Science & Technology*.

[11] Guandalini B. B. V., Rodrigues N. P., Marczak L. D. F. (2019). Sequential extraction of phenolics and pectin from mango peel assisted by ultrasound. *Food Research International*.

[12] Kobus Z., Krzywicka M., Pecyna A., Buczaj A. (2021). Process efficiency and energy consumption during the ultrasound-assisted extraction of bioactive substances from hawthorn berries. *Energies*.

[13] Tran T. T. B., Saifullah M., Nguyen N. H., Nguyen M. H., Vuong Q. V. (2021). Comparison of ultrasound-assisted and conventional extraction for recovery of pectin from Gac (Momordica cochinchinensis) pulp. *Future Foods*.

[14] ICONTEC (2022). *Fresh Fruits, Mangos Criollos: Specifications (NTC 5139)*.

[15] Esparza-Merino R. M., Macías-Rodríguez M. E., Cabrera-Díaz E., Valencia-Botín A. J., Estrada-Girón Y. (2019). Utilization of by-products of Hibiscus sabdariffa L. as alternative sources for the extraction of high-quality pectin. *Food Science and Biotechnology*.

[16] AOAC (2006). *AOAC International Official Methods of Analysis*.

[17] Wannabussapawich B., Seraypheap K. (2018). Effects of putrescine treatment on the quality attributes and antioxidant activities of ‘Nam Dok Mai No. 4’ mango fruit during storage. *Scientia Horticulturae*.

[18] Wang M., Huang B., Fan C. (2016). Characterization and functional properties of mango peel pectin extracted by ultrasound assisted citric acid. *International Journal of Biological Macromolecules*.

[19] Pradal D., Vauchel P., Decossin S., Dhulster P., Dimitrov K. (2016). Kinetics of ultrasound-assisted extraction of antioxidant polyphenols from food by-products: extraction and energy consumption optimization. *Ultrasonics Sonochemistry*.

[20] Wang Y., Qin K., Chen F. (2022). Texture improvement of fermented minced pepper under vacuum impregnation with pectin methylesterase and CaCl_2_ during fermentation. *International Journal of Food Science & Technology*.

[21] Liao J., Qu B., Liu D., Zheng N. (2015). New method to enhance the extraction yield of rutin from Sophora japonica using a novel ultrasonic extraction system by determining optimum ultrasonic frequency. *Ultrasonics Sonochemistry*.

[22] Zhang Q.-A., Shen H., Fan X.-H., Shen Y., Wang X., Song Y. (2015). Changes of gallic acid mediated by ultrasound in a model extraction solution. *Ultrasonics Sonochemistry*.

[23] Cui R., Zhu F. (2021). Ultrasound modified polysaccharides: a review of structure, physicochemical properties, biological activities and food applications. *Trends in Food Science & Technology*.

[24] Grassino A. N., Brnčić M., Vikić-Topić D., Roca S., Dent M., Brnčić S. R. (2016). Ultrasound assisted extraction and characterization of pectin from tomato waste. *Food Chemistry*.

[25] Sánchez-Camargo A. P., Montero L., Mendiola J. A., Herrero M., Ibáñez E. (2020). Novel extraction techniques for bioactive compounds from herbs and spices. *Herbs, Spices and Medicinal Plants: Processing, Health Benefits and Safety*.

[26] Pérez S., Rodríguez-Carvajal M. A., Doco T. (2003). A complex plant cell wall polysaccharide: rhamnogalacturonan II. A structure in quest of a function. *Biochimie*.

[27] Wongkaew M., Sommano S. R., Tangpao T., Rachtanapun P., Jantanasakulwong K. (2020). Mango peel pectin by microwave-assisted extraction and its use as fat replacement in dried Chinese sausage. *Food*.

[28] Freitas de Oliveira C., Giordani D., Lutckemier R., Gurak P. D., Cladera-Olivera F., Ferreira Marczak L. D. (2016). Extraction of pectin from passion fruit peel assisted by ultrasound. *LWT-Food Science and Technology*.

[29] Rodsamran P., Sothornvit R. (2019). Microwave heating extraction of pectin from lime peel: characterization and properties compared with the conventional heating method. *Food Chemistry*.

[30] Kazemi M., Khodaiyan F., Labbafi M., Saeid Hosseini S., Hojjati M. (2019). Pistachio green hull pectin: optimization of microwave-assisted extraction and evaluation of its physicochemical, structural and functional properties. *Food Chemistry*.

[31] Venzon S. S., Canteri M. H. G., Granato D. (2015). Physicochemical properties of modified citrus pectins extracted from orange pomace. *Journal of Food Science and Technology*.

[32] Cerón-Martínez L. J., Hurtado-Benavides A. M., Ayala-Aponte A., Serna-Cock L., Tirado D. F. (2021). A pilot-scale supercritical carbon dioxide extraction to valorize Colombian mango seed kernel. *Molecules*.

